# Effects of disciplinary cultures of researchers and research trainees on the acceptability of nanocarriers for drug delivery in different contexts of use: a mixed-methods study

**DOI:** 10.1007/s11051-015-2998-1

**Published:** 2015-04-17

**Authors:** Vanessa Chenel, Patrick Boissy, Jean-Pierre Cloarec, Johane Patenaude

**Affiliations:** Interdisciplinary Institute for Technological Innovation (3IT), Université de Sherbrooke, Sherbrooke, QC Canada; Department of Surgery, Faculty of Medicine and Health Sciences, Université de Sherbrooke, 3001-12th Avenue North, Sherbrooke, QC J1H 5N4 Canada; Laboratoire Nanotechnologies et Nanosystèmes (LN2), Centre National de la Recherche Scientifique (CNRS), Université de Sherbrooke, Sherbrooke, Canada; Institut des Nanotechnologies de Lyon (INL), Site École Centrale de Lyon, Université de Lyon, Lyon, France

**Keywords:** Acceptability, Acceptance, Impact perception, Nanomedicine, Researchers’ perceptions, Survey, ELSI

## Abstract

The acceptability of nanomedical applications, which have the potential to generate ethical and societal impacts, is a significant factor in the deployment of nanomedicine. A lack of fit between nanomedical applications and society’s values may result from a partial consideration of such impacts. New approaches for technological evaluation focused on impact perception, acceptance, and acceptability are needed to go beyond traditional technology assessment approaches used with nanotechnology, which focus mainly on toxicological and safety criteria. Using a new evaluative approach based on perceived impacts of nanotechnology, the objective of this study was to assess perceptions among researchers and research trainees familiar with emergent technologies and from different disciplinary background the scope of acceptability judgments made towards the use of nanocarriers. This mixed-methods study was based on scenarios presenting two types of drug-delivery nanocarriers (carbon, synthetic DNA) in two contexts of use (lung cancer treatment, seasonal flu treatment). Researchers and research trainees in the natural sciences and engineering, and the social sciences and the humanities were invited by email to take part in this project. An online questionnaire followed by semi-directed interviews allowed characterization of disciplinary divergences regarding to impact perception, acceptance, and acceptability of the scenarios. The results suggest that impact perception is influenced by disciplinary culture. Also, trends can be seen between respondents’ profiles and variables of acceptance and acceptability, and certain components of the acceptability judgement are specific to each disciplinary culture. The acknowledgment and consideration of these disciplinary divergences could allow, among others, for opening up interdisciplinary dialogue on matters related to the acceptability of nanomedical applications and their developments.

## Introduction

Advances in nanotechnologies (NT) offer promising avenues of applications across many fields but also raise important ethical, legal and social implications (ELSI) for these applications (Grieger et al. 2010; Roco and Bainbridge 2005). It is now more and recognized that omitting to take these issues into account can compromise the downstream deployment of NT (Fisher et al. 2006). Questions related to the acceptability of new applications of NT must be addressed upstream and midstream through continuous technology evaluations with the different stakeholders involved in the development, deployment and use of NT (CEST 2006). Traditional approaches of technology evaluation are centered on the concept of acceptance and tend to focus on the examination of factors linked to the prediction of the intention to use or the willingness to pay from targeted users of a technology (Siegrist et al. 2007a; Slovic 1987). Risk perception, where risk refers to the danger of death or injury, is often emphasized in such evaluations, whereas other factors valued by the public are seldom considered (Kahan et al. 2009; Sandler and Kay 2006). The tumultuous history of the worldwide production and consumption of genetically modified organisms (GMOs) is a good example of this phenomenon. Indeed, even though regulatory bodies in North America and Europe deemed the consumption of GMOs as safe, social tensions emerged when the public rejected this new technology, judging that its risks outweighed the predicted benefits (Gaskell et al. 2004; Roco et al. 2008). In this case, the members of the public perceived possible impacts of the production and consumption of GMOs on a complex set of ethical, environmental, economic, legal and social aspects, and gave these issues weight in their judgements of acceptability (Patenaude et al. 2015). An assessment approach going beyond a judgement about facts—acceptance—and tending instead towards a judgement about values—acceptability—could have enhanced the understanding of all stakeholders’ values, allowing developers of GMOs to emerge from this stalemate and better direct their development in accord with societal values. How does this apply to NT? As the bulk of research on nano materials is focused on public acceptance based on toxicological and safety criteria (Nabeshi et al. 2011; Wang et al. 2011), the same can happen. While judgement from the public will be modulated by these criteria and with the specific application of NT considered, undeniably ELSI will come into play in the judgements of acceptability. Assessment approaches incorporating those implications could help in preventing a failure between new technologies and society’s values (Bennett and Sarewitz 2006; Mnyusiwalla et al. 2003).

Historically, through scientific communication, researchers have contributed to shaping public perception and opinion on NT (Corley et al. 2011; Ho et al. 2010). In a context of participative governance, researchers’ perspective may also be used as a reference to open up discussion on the development of NT (Sahoo 2013). As researchers from numerous fields are involved in the development of NT, their perspectives regarding NT are wide-ranging and traditionally communicated through reports and publications in isolation according to the disciplinary culture. Given the range of the possible ELSI of NT applications, new approaches will have to bridge the gap between researchers’ discourse in natural sciences and engineering (NSE) and social sciences and humanities (SSH). Moreover, the judgement of experts in NSE serves no longer as a warrant of reliability for new technologies, the way it did a few decades ago. Thus, the assessment of researchers in the SSH, whose point of view brings a different perspective, must therefore be taken into account when a new technology is being developed (Denicourt 2006; Scheufele and Lewenstein 2005). With uncertainties attached to NT and present-day democratic societies no longer satisfied with the technological criteria of efficacy, efficiency, and safety as grounds for the acceptability of a technology, experts in the NSE will also be called upon to integrate this reflective approach into the process of developing new technologies in order to develop their perspective. In a context of midstream modulation, combined perspectives of researchers from both sets of disciplinary cultures might be beneficial to NT development by enhancing the richness of the debate. However, previous works on the perceptions of risks and benefits and on the acceptance of NTs have shown that researchers in the NSE involved in the development of new technologies perceive, and feel concerned by ELSI (Besley et al. 2008; Gupta et al. 2013; Siegrist et al. 2007b), even though they sometime “are unable to make direct connections between ethics and what they do” [(Berne 2006) quoted in (Bassett 2012)]. The importance of the complementary viewpoints of experts from both sets of disciplinary fields calls for recourse to interdisciplinary dialogue when addressing questions of NT acceptability. Identifying divergences between disciplinary cultures (DC) of SHE and NSE toward NT could allow for a better planning of the space for such an interdisciplinary dialogue and a reciprocal understanding of the perspectives of all the players.

The influence of scientists’ DC on the perception of risks associated with NTs has been studied by numerous authors. Some studies have revealed major variations attributable to the frames of reference embedded in the different disciplinary backgrounds (Powell 2007) and to the epistemological frameworks specific to each discipline (Althaus 2005; Lafontaine 2003). Working with varied disciplinary profiles, (Weisenfeld and Ott 2011) confirmed that DC exerts an influence on the perception of technological risk. The study took four areas of application into account (renewable energies, genetic engineering, nanotechnology, and information and communication technologies) and showed that the type of application studied influenced risk perception, a finding confirmed regarding NT applications for water and food by others (te Kulve et al. 2013). Patra et al. (2010) confirmed that the majority of NT practitioners questioned perceive ethical impacts to be related to the development of their technologies. However, no specific disciplinary difference emerged among these scientists, all of whom had backgrounds in NSE. An overview of the literature reveals that few studies have emphasized the heterogeneous nature of the status of expert in NT and that a gap remains when it comes to studying impact perception and acceptability of nanotechnological applications among players in the SSH.

Medicine is a field of application where advances in NT are likely to knock down many technological barriers, creating opportunities for new diagnostics tools and clinical interventions (Nijhara and Balakrishnan 2006). The areas of application for nanomedicine (NM), defined as medicine on the molecular scale (Freitas 2005), or as the application of NT to health care (Farokhzad and Langer 2006), are vast and range from prevention to diagnosis and treatment. NM is likely to give rise to numerous impacts on society as well as on the representations of the human being and health (Allhoff 2009). Conflicts around redistribution, justice, and equity in health care must also be considered anew in the context of NM (Allhoff 2009; Bawa and Johnson 2009). Even though it has been documented that practitioners in the field of health care perceive some ethical issues associated with the application of NTs in medicine but without recognizing them as new issues specific to NM (Silva Costa et al. 2011), experts’ impact perception in direct connection with NM has not been studied. Among all the nanomedical applications, targeted drug delivery by nanocarriers is amongst the forerunner in terms of promises and R&D efforts in NM. Advances in this sphere could broaden the range of therapeutic agents used and help in developing new approaches to direct active principles directly to the desired targets for therapy (Bawa and Johnson 2009; Hughes 2005). This could be particularly impactful in cancer therapy (Peer et al. 2007; Ranganathan et al. 2012).

Several studies have examined perceptions and acceptance of NT applications in general, but the topic of NM per se has barely been touched upon, with any deepened exploration of these concepts in relation to tangible applications. Based on a new conceptual framework (Patenaude et al. 2015), a first portion of this study has described the variables of impact perception, acceptance, and acceptability in relation to two materials (carbon nanocarrier, synthetic DNA nanocarrier) and two contexts of use (lung cancer treatment, seasonal flu treatment) in researchers (Chenel et al. 2015). It was shown that although the material from which the nanocarrier is made influences perceived impacts, it does not influence acceptance and acceptability. Context of use, on the other hand, strongly influences the responses of acceptance and acceptability towards the nanocarriers. The present work is a continuation of the same project and completes the portrait of the variables of impact perception, acceptance, and acceptability, while allowing a better understanding of the potential divergences related to DC. The primary objective of this study is to analyse the effect of DC of researchers and research trainees on their perception of impacts, their acceptance, and their acceptability in relation to two kinds of targeted drug-delivery nanocarrier in two contexts of use. The secondary objectives are to examine the relationships between these variables, in relation to respondents’ DC, and to explore acceptability judgements in relation to possible cultural divergences.

## Materials and methods

### Conceptual framework and study design

The study relies on a new theoretical framework for the analysis of the impacts and acceptability of NT proposed by Patenaude et al. (2015). This framework takes into account the considerations related to all of the ethical, environmental, economic, legal and social aspects. In addition to the nature of the considerations, this framework also allows for the targeting not only of the risks (negative impacts) but also of the benefits (positive impacts) that could flow from the development and use of new technologies. The first part of the framework relates to the perceived impacts of the technology being assessed. Impact perception is defined as a two-dimensional examination of perceived impacts, based on the estimated probability of occurrence of given impacts (identification) and on the importance assigned by a participant to each of these impacts (evaluation). A second variable integral to the framework refers to acceptance. Individual acceptance is defined as the intention by a user to use a technology or a device in a specified context of use, while social acceptance corresponds to a personal evaluation of the level of development desirable for society of a technology or a device with a specified use. Last, the deployment of the third variable integral to the framework, acceptability, allows for going beyond the simple fact of acceptance and consists of a weighting of the technology’s or device’s impacts on certain priority issues, in order to arrive at a value judgement about what is acceptable. Individual acceptability refers to the value judgment regarding all the impacts that accounts for individual acceptance, while social acceptability refers to the value judgment regarding all the impacts that accounts for the evaluation of the desirable level of development. Using this conceptual framework, a two phase mixed-methods design (quantitative phase with web-based questionnaire, qualitative phase with semi-directed interviews) with a sequential data triangulation (QUANTITATIVE → qualitative) was chosen to develop multiple perspectives and a complete understanding of the research objectives proposed. An Institutional Review Board of the Centre Hospitalier Universitaire de Sherbrooke (CHUS) approved the two phases of the study and participants gave their consent to participate.

### Study participants and recruitment

#### Web-based questionnaire

The recruitment strategy for the study participants in the quantitative phase was based on the identification through a exhaustive literature review and on-line search using keywords such as *nanotechnology*, *nanomedicine*, *ethics*, *social sciences,* and *new technologies of* authors publishing on topics associated with new technologies and or individuals having affiliations with research groups, labs, or networks that conduct research on the new technologies (for example, the NE^3^LS Network on Nanotechnology (NE^3^LS NetWork 2014)—Canada and Pacte—Social Science Research Laboratory (PACTE 2014)—France). As the questionnaire was developed and tested in French, for reasons related to language, only Francophone researchers and research trainees were targeted. To obtain the largest possible number of respondents, recruitment was conducted among researchers and research trainees in Canada and Europe. A list of 1527 researchers and research trainees (graduate students) was generated as potential participants—the term *researcher* will be used generically from this point to describe all participants. An email inviting the recipient to fill out the web-based questionnaire was sent to the targeted researchers (*n* = 1320 valid invitations, 230 invalid emails) in September 2013, followed by two reminder emails.

#### Semi-directed interviews

At the end of the web-based questionnaire, researchers who were interested in taking part in the qualitative phase were invited to provide their contact information—stored separately from the other data by email. From December 2013 to April 2014, the semi-directed interviews were conducted with a final subsample of about 10 % of the researchers who had completed the quantitative phase (*n* = 22). Participants in the qualitative phase were chosen based on their DC and their geographical location to ensure a representative sample of participants recruited in the quantitative phase. Interviews lasted on average one hour and were conducted in person (*n* = 15), or by means of a teleconferencing or videoconferencing system (*n* = 7).

### Instruments and variables

#### Quantitative phase

The study’s quantitative phase was in the form of a web-based questionnaire based on the operationalization of the conceptual framework designed by Patenaude et al. (2015). The variables of impact perception, acceptance, and acceptability were considered through the optics of a scenario-based approach. Scenarios related to the use of two kinds of drug-delivery nanocarrier (carbon nanocarrier, synthetic DNA nanocarrier) in two contexts of use (lung cancer treatment, seasonal flu treatment) were presented to the participants. Six major positive and negative impacts (drawn from a review of the literature) on issues of health, the environment, and social cohabitation associated with NM were then presented to participants. Impact perception was measured by combining the respondent’s estimation of the probability that a given impact would arise and the importance assigned by the respondent to each such impact. This yielded a perception index (PI) that might be negative, neutral, or positive. Individual acceptance (IndAtce) was measured based on the respondent’s intention to use each type of treatment in each of the two clinical contexts. Social acceptance (SocAtce) was based on the level of development for the treatment that the respondent deemed desirable for society. Acceptability was measured by establishing a weighting of the positive and negative impacts perceived and prioritized by the respondent in arriving at the decision regarding personal use or in arriving at the level of development deemed desirable for society. This yielded an individual acceptability index (IndAI) and a social acceptability index (SocAI) that both might be negative, neutral, or positive. The questionnaire included two subvariables of acceptability, namely preponderant issue (IndPIssue, SocPIssue) and perceived usefulness (Useful/Ind, Useful/Soc). The preponderant issue offers a portrait of the issue or issues prioritized in arriving at the individual and social judgements of acceptability. This variable also highlights whether a single issue (health, the environment, social cohabitation) was prioritized by respondents or whether in contrast it was a combination of issues that characterized respondents’ judgements of individual (IndPIssue) and social (SocPIssue) acceptability. Perceived usefulness measures the extent to which a respondent deemed each kind of nanocarrier in each clinical context to be useful to himself/herself and to society. This variable does not constitute an integral part of the conceptual framework, but is a constituent variable in traditional Technology Assessment Models (TAM) (Davis 1989). All variables were measured with four-point Likert scales (for more detail, see Chenel et al. 2015).

Before being posted online, the questionnaire was pretested by means of cognitive interviewing (Willis 2004). Participants (*n* = 35) who were representative of the population under study were recruited and asked to complete the questionnaire in person while we observed them and an interviewer debriefed them after each question. Three cycles of interviews to optimize the questionnaire components (instructions, key concepts, scenario presentation, questions and response options) and test the robustness and usability (completion time, ease of administration, visual aspect) of the on-line version were conducted and changes to the questionnaire were made after each cycle. In the last round, an average completion time was calculated to serve as a quality control measure for the questionnaires completed during the study.

#### Qualitative phase

The same scenarios presented in the questionnaire for the use of the drug-delivery nanocarriers were also presented to participants in the qualitative phase. An interview guide was developed using preliminary analysis of the quantitative data collected in the quantitative phase to explore specific theme in the semi-directed interviews. The interview guide was focused on the components of the acceptability judgment as presented in the questionnaire but in a more open and flexible manner and without imposing limits on the choice of answers. Probes from the interviewer were added to allow the exploration of the various facets of acceptability by placing in relation to each other the two contexts of use, the two kinds of nanocarrier, notions of usefulness and effectiveness, and the reasons why a respondent’s judgement might be modulated in a given situation.

### Data analysis

#### Statistical analysis of quantitative data

The influence of DC on the variables of impact perception, acceptance, and acceptability, in relation to the different kinds of nanocarrier and the two contexts of use, were tested using the Mann–Whitney *U* test and the Pearson Chi square test for independence. A multiple correspondence analysis (MCA) was performed in order to examine relationships between core variables and respondents’ profiles. This analysis, which is specific to categorical variables (and part of a family of descriptive methods that includes clustering and factor analysis and principal components analysis), reveals patterning in complex datasets and enables the visualization of independent clusters on (usually) a two-dimensional plane (Greenacre 2007). All statistical tests used an alpha of 0.05. Analyses were performed using SPSS Statistics v20. (IBM Corporation, Armonk, NY).

#### Thematic content analysis of qualitative data

Individual interviews were audio-recorded and then transcribed verbatim with no reformulation by a third party. The verbatim were read while playing back the audio recordings to ensure adequacy of transcriptions and familiarise with the themes handled in each interview. A thematic content analysis approach was used to objectively and systematically capture the discourse of the researchers interviewed (Berelson 1971). A mixed coding method based on the theoretical framework guided the identification, reviewing, and classification of the interview’s components (Paillé and Mucchielli 2012). Coding was conceptualized based on general themes from the major categories of existing issues described in the framework, but was also open to the emergence of code related to the theme of acceptability. Analysis was based on the occurrence of themes in each researcher’s discourse, rather than on the frequency of themes’ occurrence. This allowed for examining the recurrence of these themes within each DC and for highlighting divergences in the remarks made by the groups under study (Mucchielli 1979). Analyses were performed by VC using the qualitative data analysis software, Dedoose v4.12 (SocioCultural Research Consultants, UCLA, CA).

## Results

For the quantitative phase, 1320 researchers were contacted, 585 accessed the questionnaire (44.32 % access rate), and 214 completed it satisfactorily (16.21 % response rate meeting quality criteria). Of all respondents (*n* = 214), 71 % identified themselves as researchers and 29 % as research trainees. Sixty-seven percent of respondents were in the fields of the NSE and the rest in the fields of the SSH. Europeans accounted for 66 % of respondents (France = 58 %; Belgium = 5 %; Switzerland = 2 %; Italy = 1 %). Men accounted for 63 % of the sample. For the study’s qualitative phase, the final subsample (*n* = 22) included European researchers (*n* = 11) in NSE (*n* = 6) and SSH (*n* = 5) and Canadian researchers (*n* = 11) in NSE (*n* = 5) and SSH (*n* = 6) from diverse disciplinary background (see Table 1).

**Table 1:** Brief profile of researchers and research trainees

SI. No.	Disciplinary culture	Disciplinary background	Social culture
QSNF03	NSE	Biology—nanomedicine	France
QSNF02	NSE	Chemistry—nanomaterials	France
QSNF01	NSE	Chemistry—nanosensors	France
QSNQ03	NSE	Chemistry engineering—nanotechnology	Canada
QSNQ01	NSE	Electric engineering—nanotechnology	Canada
QSNQ05	NSE	Electric engineering—nanotechnology	Canada
QSNF05	NSE	Informatics—biotechnology	France
QSNF04	NSE	Medicine—radiation oncology	France
QSNQ02	NSE	Microbiology—nanosensors	Canada
QSNF06	NSE	Nanomedicine—biomimicry	France
QSNQ04	NSE	Process chemistry	Canada
QSHSQ04	SSH	Applied ethics—neuroethics	Canada
QSHSQ02	SSH	Bioethics—clinical research	Canada
QSHSQ01	SSH	Bioethics—epigenetics	Canada
QSHSQ05	SSH	Ethics—anthropology	Canada
QSHSQ06	SSH	Ethics—technological innovation	Canada
QSHSF03	SSH	Human factors and ergonomics	France
QSHSF02	SSH	Philosophy—applied Ethics	France
QSHSF05	SSH	Philosophy—applied Ethics	France
QSHSQ03	SSH	Philosophy—applied Ethics	Canada
QSHSF04	SSH	Physics—ethics of nanotechnology	France
QSHSF01	SSH	Sociology of sciences	France

*NSE* Natural sciences and engineering, *SSH* social sciences and humanities, *SI*. *No*. subject identification number

### Comparisons between nanocarriers and contexts of use in relation to disciplinary culture

A comparison of perception indexes (PI) reveals the influence of DC on impact perception for the two kinds of nanocarriers. The results show that NSE researchers have a greater perception of positive impacts than do SSH researchers (*p* < 0.01 Mann–Whitney *U* test), for both the carbon nanocarrier (31.47 vs. 18.31 %) and the synthetic DNA one (44.76 vs. 25.35 %). SSH researchers have a greater perception of negative impacts than do NSE researchers (*p* < 0.01 Mann–Whitney *U* test), for both the carbon nanocarrier (46.48 vs. 27.27 %) and the synthetic DNA one (40.85 vs. 23.08 %) (see Table 2).

**Table 2:** Comparisons between nanocarriers among perception index, acceptance, and acceptability across disciplinary cultures

	Carbon		Significance	Synthetic DNA		Significance
	NSE (%)	SSH (%)		NSE (%)	SSH (%)	
PI comparisons across nanocarrier compositions						
PI						
Positive	31.47	18.31	*p* < 0.01	44.76	25.35	*p* < 0.01
Neutral	41.26	35.21		32.17	33.80	
Negative	27.27	46.48		23.08	40.85	
Acceptance and acceptability of a drug-delivered treatment for lung cancer						
IndAtce						
Accept	93.01	94.37	*p* = 0.705	93.01	85.92	*p* = 0.093
Not accept	6.99	5.63		6.99	14.08	
IndAI						
Positive	80.42	67.71	*p* = 0.060	81.82	64.79	*p* < 0.01
Neutral	11.19	23.94		9.09	19.72	
Negative	8.39	8.45		9.09	15.49	
SocAtce						
Accept	92.31	92.96	*p* = 0.865	92.31	85.92	*p* = 0.139
Not accept	7.69	7.04		7.69	14.08	
SocAI						
Positive	78.32	59.15	*p* < 0.01	81.12	56.34	*p* < 0.01
Neutral	12.59	32.39		11.19	29.58	
Negative	9.09	8.45		7.69	14.08	
Acceptance and acceptability of a drug-delivered treatment for seasonal flu						
IndAtce						
Accept	20.98	15.49	*p* = 0.337	20.28	18.31	*p* = 0.733
Not accept	79.02	84.51		79.72	81.69	
IndAI						
Positive	25.17	21.13	*p* = 0.945	25.87	19.72	*p* = 0.364
Neutral	19.58	25.35		23.08	23.94	
Negative	55.24	53.52		51.05	56.34	
SocAtce						
Accept	23.78	14.08	*p* = 0.099	22.38	16.90	*p* = 0.351
Not accept	76.22	85.82		77.62	83.10	
SocAI						
Positive	29.37	19.72	*p* = 0.878	30.77	25.35	*p* = 0.639
Neutral	21.68	36.62		23.78	28.17	
Negative	48.95	43.66		45.45	46.48	

*PI* Perception index, *IndAtce* individual acceptance, *IndAI* individual acceptability index, *SocAtce* social acceptance, *SocAI* social acceptability index, *NSE* natural sciences and engineering, *SSH* social sciences and humanities

With regards to acceptance, no significant disciplinary difference (*p* > 0.05 Pearson Chi square independence test) was observed in the scores for the variables of IndAtce and SocAtce for the two kinds of nanocarrier. This was the case for both contexts of use. Researchers from both sets of disciplinary backgrounds accepted personal use of the carbon nanocarrier (rates of acceptance: NSE = 93.01 %, SSH = 94.37 %) as well as the synthetic DNA one (rates of acceptance: NSE = 93.01 %, SSH = 85.92 %) to treat lung cancer. On the other hand, for the treatment of seasonal flu, respondents were hesitant about personal use of both the carbon nanocarrier (rejection rates: NSE = 79.02 %, SSH = 84.51 %) and the synthetic DNA one (rejection rates: NSE = 79.72 %, SSH = 81.69 %). Similar results were obtained regarding social acceptance.

As for the acceptability index (AI) in the context of lung cancer treatment, a comparison between DCs reveals significant differences (*p* < 0.01 Mann–Whitney *U* test). Researchers from NSE fields appeared to have based on their judgements of acceptability on positive impacts to a greater extent than did SSH researchers. For individual acceptability, this was the case for the synthetic DNA nanocarrier to treat lung cancer (NSE = 81.82 %, SSH = 64.79 %). The findings about the social acceptability for the lung cancer treatment were similar for the carbon nanocarrier (NSE = 78.32 %, SSH = 59.15 %) as well as for the synthetic DNA one (NSE = 81.12 %, SSH = 56.34 %). No cultural difference was observed for acceptability in using a seasonal flu treatment.

**Fig. 1:**
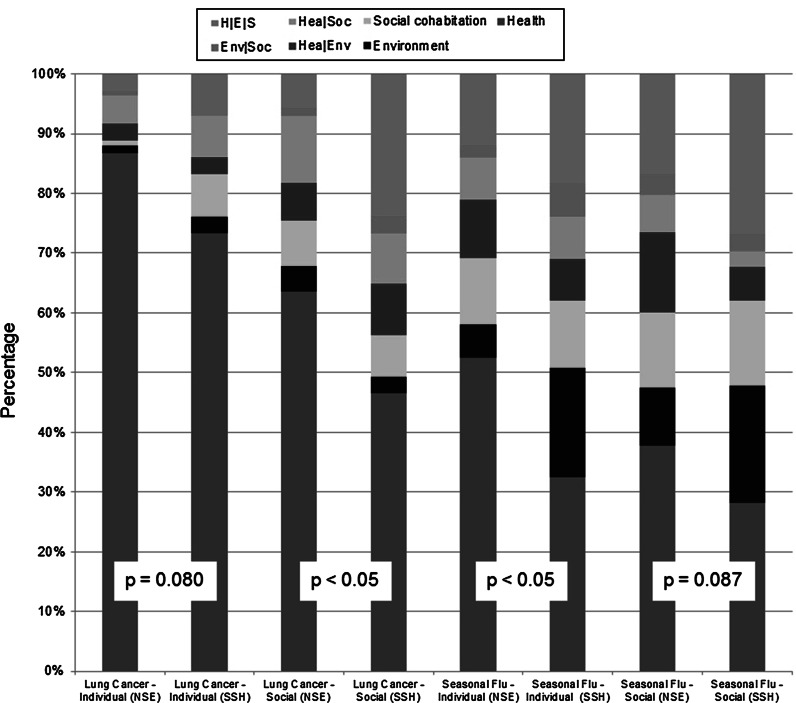
Comparisons of individual and social preponderant issues in relation to carbon nanocarrier among contexts of use, as related to disciplinary cultures. *Abbreviations* H | E | S = complex profile where all issues are equally preponderant, Env | Soc = complex profile where environmental and social cohabitation issues are preponderant, Hea | Soc = complex profile where health and social cohabitation issues are preponderant, Hea | Env = complex profile where health and environmental issues are preponderant. *NSE* natural sciences and engineering, *SSH* social sciences and humanities

An examination of PIssue relating to the carbon nanocarrier reveals certain cultural differences (*p* < 0.05 Chi square independence test) when it comes to the issues prioritized in the acceptability judgement (see Fig. 1). Cultural differences were noted as regards the IndPIssue for the seasonal flu treatment as well as the SocPIssue for the lung cancer treatment. NSE researchers appear to have based on their acceptability judgements against the personal use of seasonal flu treatment on health issues (NSE = 52.4 %, SSH = 32.4 %), while SSH researchers also significantly emphasized environmental issues in accounting for their rejection (NSE = 5.6 %, SSH = 18.3 %). Concerning the social acceptability of the lung cancer treatment, NSE researchers based their favourable judgement on health issues (NSE = 63.6 %, SSH = 46.5 %), while SSH researchers assigned importance to environmental and social cohabitation issues as well. On the other hand, for lung cancer treatment, no cultural differences relating to IndPIssue were observed, with health being prioritized by researchers from both sets of disciplinary backgrounds (NSE = 86.7 %, SSH = 73.2 %); nor were cultural differences observed at the social level for the treatment of seasonal flu, with all issues (health, the environment, social cohabitation) being emphasized in a more evenly distributed manner.

### Relationships between impact perception, acceptance, and acceptability in relation to researchers’ profiles

An examination of the relationships among variables and respondents’ profiles was carried out by including in the model disciplinary culture (NSE, SSH), social culture (European, Canadian), sex, and occupation (researcher, research trainee) in addition to PI, IndAtce, SocAtce, IndAI, SocAI, Useful/Ind and Useful/Soc. The analysis was performed for the scenario that had elicited the greatest variation in responses, namely the carbon nanocarrier to treat seasonal flu.

**Fig. 2:**
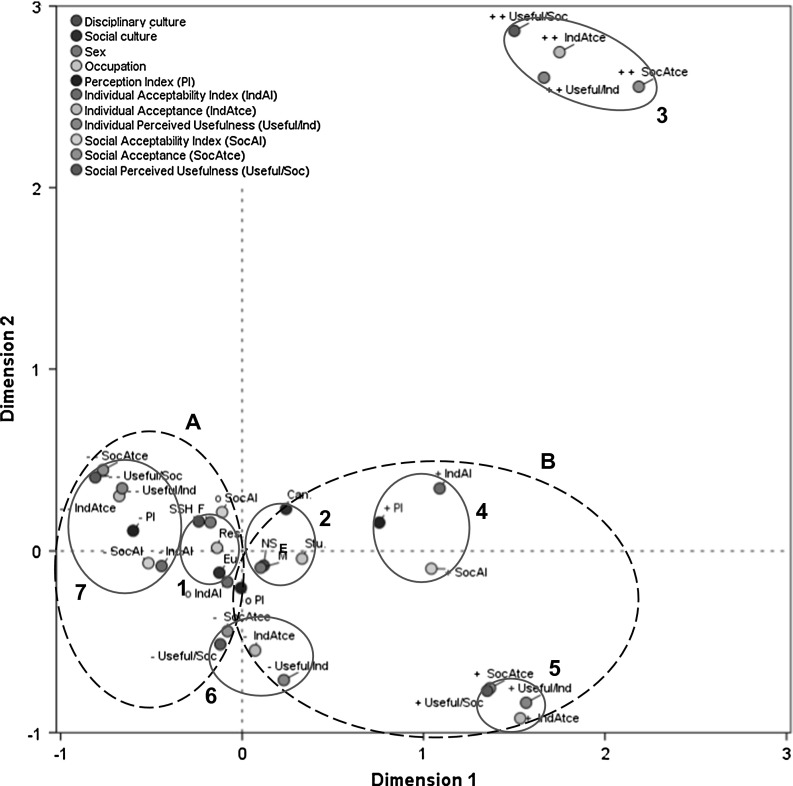
Multiple correspondence analysis: coordinates graph of core variable modalities, including individual and cultural factors. *Modalities* − = Negative, o = Neutral, + = Positive for PI, IndAI, and SocAI; − − = Wholly disagree, − = Somewhat disagree, + = Somewhat agree, ++ = Wholly agree for IndAtce, Useful/Ind, SocAtce; Useful/Soc; *NSE* natural sciences and engineering, *SSH* social sciences and humanities; *Eu.* Europe, *Can.* Canada; *M* male, *F* female; *Stu.* research trainee (graduate student), *Res.* researcher

Performing a MCA on all the data (*n* = 214) relative to the chosen scenario reveals a total explained inertia of 63.3 %, of which 36.9 % is attributable to dimension 1, corresponding to the orientation (positive/negative) of modalities, and 26.2 % is attributable to dimension 2, corresponding to the polarization (low/high) of modalities. Cronbach’s alpha (*α* = 0.783) indicates satisfactory consistency for all measured items. Only IndAtce (*D*1 = 71.4 %; *D*2 = 67.9 %), UsefulInd (*D*1 = 65.8 %; *D*2 = 68.1 %), SocAtce (*D*1 = 75.9 %; *D*2 = 58 %), and UsefulSoc (*D*1 = 72.7 %; *D*2 = 80.6 %) presented strong correlations with dimension 1 and dimension 2. All other variables were weakly correlated with both dimensions. A visualization of the MCA results is presented in Fig. 2. The graph coordinate reveals seven clusters. Clusters 1 and 2 bring together respondent profiles that share certain characteristics in relation to the variables under study. Thus cluster 1 groups together researchers, individuals from SSH fields, women, and Europeans; while cluster 2 groups together research trainees, individuals from NSE fields, Canadians, and men. Clusters 3, 5, 6, and 7 illustrate the proximity of the modalities for the variables IndAtce, SocAtce, UsefulInd, and UsefulSoc, testifying to a strong relationship among these variables. Clusters 4 and 7 illustrate the proximity between the modalities for the variables PI, IndAI, and SocAI, attesting in this instance to a less strong relationship among these variables.

The results of this analysis do not allow for pinpointing clear relationships between respondents’ profiles and the variables under study. Two metaclusters, however, do allow for drawing a link between each profile and a general trend. Metacluster A incorporates the profile of respondents associated with cluster 1 (researchers, SSH, women, Europeans) and assimilates it to those diagram modalities that are negatively oriented to dimension 1. Cluster 1 thus presents a profile of respondents that are more likely to resist using the carbon nanocarrier to treat the flu, based on an emphasis on the negative impacts. On the other hand, metacluster B incorporates the profile of respondents associated with cluster 2 (research trainees, NSE, men, Canadians) and assimilates it to those diagram modalities that are positively oriented to dimension 1. Cluster 2 thus presents a profile of respondents that are more likely to favour use of the carbon nanocarrier to treat the flu, based on an emphasis on the positive impacts.

### Exploration of value judgments of acceptability

#### Exploration of the acceptability of the two nanocarriers in different contexts of use

The results of the quantitative phase showed that the composition of the drug-delivery nanocarriers (carbon, synthetic DNA) did not have a bearing on the intention to use the treatments described, whatever the respondent’s DC. The interviews made it possible to investigate this finding further and attain a deeper understanding of the reasons for this indifference regarding the composition of the nanocarrier. An initial theme, focused on the importance of the medication’s safety and on its clinical effects, may be a part of the answer. Here is how one participant presents it:

If you bring an anti-cancer chemotherapy molecule into the tumour zone, what counts is the efficacy of the chemotherapy, the chemotherapy molecule that’s brought there, more than the capsule that brings it there. We assume both (kinds of nanocarrier) do the same work as carriers. | QSNF04

This theme was raised by half the participants. A disciplinary divergence did emerge, according to which this indifference regarding the composition of the nanocarrier was prevalent among NSE researchers and less present among SSH researchers. Another aspect of this indifference to the composition of the nanocarrier in the context of use has to do with the interviewees’ self-perception as experts. In a context of sickness, they find themselves playing the role of patient, with no real ability to form a judgement that matches the competency of the attending physician. Here is a quotation that illustrates this reality:

When you go to the hospital as a patient, from the moment you’re undressed and clothed in the patient gown, you go from the status of normal individual to the status of patient. So you enter into a relationship that I’d call, in quotation marks, one of “inferiority” to the physicians, nurses, the medical staff, who have the knowledge and experience. So since you don’t have the medical competence, you’re obliged to trust in their competence. And since, besides, to a greater or lesser extent they have your life in their hands, well, you trust them. | QSNF03

It would thus appear that a second theme, focused on the physician’s expertise and on confidence in the medical staff, could account for the fact that, for some respondents in both sets of disciplines, it seems unreasonable to express a personal preference regarding a detail as precise as the composition of the nanocarrier—which is not itself the active agent—for a medical treatment.

In contrast, the clinical context seems to be a source of great variation in terms of acceptability among the researchers. While the composition appears to have had no impact on acceptance or acceptability regarding the treatments presented in the scenarios, the interviews did reveal that the context of use played a part in the intention to use a treatment. In entering more deeply into this question, some key elements allowed for an understanding of these distinctions in relation to acceptance and acceptability for lung cancer treatment and flu treatment. An examination of acceptability judgements revealed different viewpoints on the acceptability of nanocarriers depending on context of use. Even though more impacts have been mentioned by researchers, Table 3 presents distinctions in impacts invoked in arriving at acceptability judgement regarding the two context of use, regardless to disciplinary cultures. Specifically, with the appearance of a new kind of treatment, problems of access and equity were highlighted in the context of lung cancer treatment. The possibility of increased efficacy in the treatment of this fatal disease, and increased life expectancy, was two more factors that were more often adduced, out of the seven that were raised as explanations for the acceptability of nanocarriers in treating lung cancer. When it came to treating the seasonal flu, five factors were adduced. It was mentioned that a new effective treatment for the seasonal flu could contribute to reducing the disease’s social impact by cutting down on transmission. On the other hand, the development of a treatment of high complexity—using nanocarriers—was significantly challenged:

I think that with that, you’re really using a bazooka to kill a fly. Okay, this (i.e., nanocarriers), this isn’t a bazooka, but I think it’s disproportionate, too big. | QSNF04

**Table 3:** Distinctions in impacts invoked in arriving at acceptability judgement regarding context of use of targeted drug-delivery nanocarriers

Issue	Impact	Context of use
Health	Resulting undesirable effects	**Lung cancer** > Seasonal flu
	Resulting desirable effects	**Lung cancer** > Seasonal flu
	Improved well-being	**Lung cancer** > Seasonal flu
	Disturbance of body’s homeostasis	Lung cancer < **Seasonal flu**
Life and death	Treatment of a potentially fatal disease	**Lung cancer** > Seasonal flu
	Improved life expectancy	**Lung cancer** > Seasonal flu
Social cohabitation	Accessibility issues/inequalities	**Lung cancer** > Seasonal flu
	Reduced impact of the disease on society	Lung cancer < **Seasonal flu**
	Increased productivity of sick people	Lung cancer < **Seasonal flu**
Environment	Increased environmental pollution	**Lung cancer** > Seasonal flu
Economy	Higher treatment costs	Lung cancer < **Seasonal flu**
Technoscience	Questioning of treatment	Lung cancer < **Seasonal flu**

In bold, the context of use for which the impact has been mainly invoked

Other contextual factors emerged from the interviews and allowed to document these differences related to the contexts of use. The gravity of the disease and the possibility of harmful consequences to health, as compared with the benign nature and the absence of significant consequences, are factors that can contribute to contemplating taking a treatment or not. The perceived seriousness of lung cancer in contrast to the seasonal flu led the majority of respondents to accept a treatment for the former and categorically refuse treatment for the latter. This passage illustrates the point:

In caring for cancer, what’s at stake is patient survival. In caring for the flu, the stakes very rarely consist of patient survival and treatments already exist. | QSSHF01

Nevertheless, some researchers have considered the treatment for the seasonal flu to be sometime desirable where a real risk to health was perceived. Although the notion of seriousness was raised by a large number of participants when all cultures are taken together, a nuance related to giving consideration to populations at greater risk in connection with less serious diseases was raised more often by SSH researchers than NSE researchers. Finally, the purpose of the treatment proposed for a given use also appears to weigh in the balance in arriving at a judgement of acceptability. A comparison between a product aimed at contributing to patient comfort and symptom reduction and a product aimed at treating and curing the patient allowed for an understanding of the variations. The perceived goal of the treatment, as invoked in this dichotomy between necessity and mere comfort, appears to play a role in arriving at a judgement of acceptability for the use of a treatment in a given context.

#### Exploration of judgement of a scenario’s acceptability in relation to disciplinary culture

The in-depth examination of the acceptability towards a single scenario, that of the carbon nanocarrier to treat seasonal flu, made it possible to bring into relief some particularities of researchers’ judgement of acceptability as related to their DC. To flesh out the differences observed based on quantitative data analysis, a thematic content analysis brought out the main issues and impacts associated with the framing of researchers’ acceptability judgements for this scenario. A list of 15 items related to issues of health, life and death, social cohabitation, the economy, the environment, representation of the human being and technoscience describes all the factors invoked by the interviewees and explaining why they accepted or not the treatment proposed (see Table 4). A different weighting for the positive and negative impacts was observed as between respondents who accepted the treatment and those who rejected it. Several factors were common to all the researchers, but cultural differences were observed in relation to seven of the 15 factors invoked in the acceptability judgement.

**Table 4:** Identification of disciplinary differences in impacts invoked in arriving at acceptability judgement regarding use of carbon nanocarrier to treat seasonal flu

Issue	Impact	Disciplinary culture
Health	Resulting undesirable effects	NSE = SSH
	Resulting desirable effects	NSE = SSH
	Disturbance of body’s homeostasis	NSE = SSH
Life and death	Treatment of a potentially fatal disease	NSE < **SSH**
Social cohabitation	Accessibility issues/inequalities	**NSE** > SSH
	Reduced impact of the disease on society	NSE = SSH
	Increased productivity of sick people	NSE < **SSH**
	Higher social burden of treatment	NSE < **SSH**
	Possibility of choosing to be treated	NSE < **SSH**
Environment	Increased environmental pollution	NSE < **SSH**
Economy	Higher development costs	**NSE** > SSH
	Higher treatment costs	NSE = SSH
	Benefits for national market	NSE = SSH
Representation of the human being	Transformed definitions of health/sickness	NSE = SSH
Technoscience	Questioning of treatment	NSE = SSH

*NSE* Natural sciences and engineering, *SSH* social sciences and humanities

Bold indicates the DC that invoked the impact more often in arriving at an acceptability judgment about treatment for the seasonal flu

Natural sciences and engineering (NSE) researchers had a more marked tendency to broach themes related to the high cost of developing such a specialized technology, and to the inequalities likely to be created by use of the treatment. SSH researchers, for their part, emphasized impacts on social cohabitation. They spoke more about the social burden the adoption of this treatment would represent, and about the importance of being able to choose to be treated with this method or to refuse it—attesting to the importance of making choice possible for all members of society, even if they personally do not agree with the use of the treatment. They also broached gains in productivity for patients treated. The interpretation of this last factor may be both positive and negative. From one perspective, the sickness of someone with an important role could have negative impacts for society. For example, a researcher suggested that if a surgeon must take prolonged leave, this could lead to negative consequences for the patients. From another perspective, a researcher’s way of looking at his/her own sickness allows to see how the weighting given to the values of self-respect and productivity as a worker can shift when the patient is at the centre of the situation:

Sometimes I wonder if the reason we want to solve a problem like the flu so fast, with this kind of medication, isn’t because we’re concerned about human health but because we’re concerned about worker productivity. And that represents a moral problem for me, because I consider myself a human being first, a citizen next, and a worker after that. So I want people to take care of my humanity, then my citizenship, and then after that let me work and not put me back to work as fast as possible if I have a health problem. | QSSHQ06

Social sciences and humanities (SSH) researchers also emphasized two factors discussed earlier, namely the possibility of increasing environmental pollution using this kind of treatment, as noted in the analysis of preponderant issues, and the importance of curing a potentially fatal disease, attesting to their sensitivity towards more vulnerable populations.

## Discussion

This study’s objectives were pursued in two phases. First, the quantitative phase was designed to examine the impact that researchers’ DC could have on variables under study, namely impact perception, acceptance, and acceptability, in relation to two kinds of targeted drug-delivery nanocarrier in two contexts of use; and to identify possible relationships between respondent profiles and the variables in question. Next, the qualitative phase was designed to shed light on certain results of the quantitative sequence and to explore acceptability judgements by scenario in relation to disciplinary divergences.

The results show that even while controlling for DC, impact perception was the only variable on which nanocarrier composition (carbon, synthetic DNA) had an effect, in contrast to acceptance and acceptability for which the nanocarrier composition did not appear to be a factor of influence. Even though impact perception is a notable covariate of acceptance and acceptability, this points out this variable’s inadequacy as a determinant of the two others and highlights the importance of incorporating other factors and contextual considerations into approaches to acceptance and acceptability. That is, results show differences in DC in relation to impact perception for the two kinds of nanocarriers. SSH researchers had a greater perception of the negative impacts of the two kinds of nanocarrier than NSE researchers who, for their part, perceived more positive impacts. This result is in line with what was observed by others in the literature. Indeed, DC, via the knowledge base acquired over the course of the exercise of one’s responsibilities (Powell 2007) and the epistemological grounding specific to each discipline (Lafontaine 2003), has been shown to influence the way a nanotechnological application is perceived. However, whether a carbon nanocarrier treatment is viewed as interchangeable with a synthetic DNA one (or vice versa) in a given context of use appears to depend on preconceptions about the two materials. In interviews, the NSE researchers, perceiving to a greater extent the positive impacts of the two kinds of nanocarrier, were less likely to show concerns about the interchangeability of the two kinds of nanocarrier for a given context of use.

On the other hand, while composition may have no effect on acceptance or acceptability, context of use does need to be taken into account. Several studies have shown the influence of the nature of a technology and the context on impact perception and acceptance (Gupta et al. 2013; te Kulve et al. 2013; Weisenfeld and Ott 2011). But so far, no one has highlighted the influence of context of use on acceptance and acceptability in relation to a NM treatment. This study has confirmed that the context of use is a factor influencing the variables under study, even though few differences emerged that were specific to DC. As regards acceptance, this may be explained by the fact that the contexts of use were quite polarizing and thus not highly conducive to cultural variation. The great gap between lung cancer, perceived as catastrophic and uncontrollable, and the seasonal flu, seen as more ordinary and subject to control, could account for greater acceptance in relation to the former and lesser acceptance in relation to the latter (Slovic 1987). The contrast between the serious nature of cancer and the benign nature of the flu emerged during interviews as a determining characteristic in the acceptance of a treatment under certain conditions. Additionally, even as confidence in government authorities would appear to govern risk perception about NT (Siegrist et al. 2007b), the emergence of the themes of trust in the physician and in medical expertise suggests that in the clinical context, when it comes to acceptance, the interviewees immediately adopt the position of user and deploy arguments suited to this position, without reference to their cultural profiles. However, certain cultural differences were observed when it came to the acceptability index and to the profile of the preponderant issue as regards cancer treatment. SSH researchers were more inclined to balance positive with negative impacts, unlike NSE researchers, who were more inclined to justify their positions by reference to positive impacts. The greater importance assigned by SSH researchers to the uncertainties and the unforeseeable long-term effects on both human health and the environment and the lesser importance assigned to the beneficial effects associated with the curing of cancer, compared to NSE researchers, could account for this cultural variation. This brings forward the possibility that researchers in the two different disciplinary spheres harboured different concerns.

As for relationships, the MCA, which incorporated both respondent profiles and the variables under study, revealed associations among all those variables. Notably, impact perception and acceptability yielded similar patterns of distribution, which indicates a degree of correspondence between these variables. While this result fits the contexts presented, there is a conceptual difference between *perceived* impacts and those that are *taken into account and prioritized* during a decision-making process (Patenaude et al. 2015). Impacts could be perceived for certain specific use situations (tobacco use, malnutrition), whereas an acceptability judgement regarding these use behaviours could be based on different arguments. The questionnaire’s operationalization, where the same list of impacts was used for measuring both variables, may have contributed to the strength of this association. Another relationship between acceptance and perceived usefulness also emerged through the MCA and was confirmed during the interviews by respondent remarks that weighed the necessary nature of a treatment for a given condition. Perceived usefulness has been documented in the literature as an important factor in accounting for information technologies’ acceptance (Davis 1985; Venkatesh and Bala 2008). Results suggest the transferability of this finding to nanomedical applications. The respondent profiles (NSE, SSH) were not associated with any of the variables under study. However, possible comparisons regarding the orientation of modalities of some variables highlight certain tendencies for the profile that includes NSE researchers to appear more optimistic about the scenario presented in opposite to the profile including SSH researchers.

An in-depth examination of researchers’ acceptability regarding the scenario of carbon nanocarrier treatment for the seasonal flu enabled exploration of the balance of perceived impacts in arriving at a judgement of acceptability. From a quantitative viewpoint, analysis of the acceptability index revealed that the majority of researchers invoked negative impacts in accounting for their acceptance. An analysis of the preponderant issue profile, however, yielded the conclusion that, in line with results shown by others (Althaus 2005; Powell 2007; Weisenfeld and Ott 2011), the factors invoked in relation to the acceptability judgement by researchers from the two DC differed. For instance, environmental pollution was a significant concern for SSH researchers but was less so for NSE researchers. The thematic content analysis of the interview transcripts brought out other factors underlying cultural differences. For example, NSE researchers invoked the high cost of development as an argument in support of a more negative acceptability judgment towards use of carbon nanocarriers to treat the seasonal flu. This also applies to the importance of making the treatment available to all, an argument invoked by SSH researchers in favour of the freedom of choice to those who wish to benefit from it.

The divergences noted between researchers from the two sets of disciplinary backgrounds underline the importance of taking account of the depth of the acceptability judgement and the nature of the issues and impacts prioritized. Since acceptability judgements are likely to be influenced by the profile of the assessor, there is an inherent interest in taking the study of acceptability to a deeper stage and understanding the impacts and issues acceptability judgements are based on, in addition to the elements that may modulate this judgement. The thematic analysis of the interview contents revealed several impacts that were identified by respondents during the interviews but were not included in the web-based questionnaire for practical reasons (cognitive load, completion time). These include, for example, the high cost of treatment to the user and concerns about the development of treatments. The absence of some of these impacts from the questionnaire could account for the fact that certain differences failed to emerge in the processing of the data from the quantitative phase but became apparent during the interview content analysis. The frequent occurrence of themes not broached in the questionnaire but identified during the interviews points to the importance of taking a more exhaustive approach in studying impact perception and acceptability.

As regards the strengths and limitations of this study, the credibility and reliability of the final results were enhanced by the use of standards such as completion time for the questionnaire results and by the use of an interview guide in conducting the interviews. A high degree of convergence was observed between the quantitative and the qualitative results, in particular with regards to acceptance and acceptability, providing partial substantiation of the validity of the pretested questionnaire. An understanding of the disciplinary differences related to acceptability that emerged in the analysis of the quantitative data was deepened through the interview process and through triangulation offered an expanding portrait of these cultural distinctions. However, considering the novelty of the framework underlying the development of the questionnaire and even though a high degree of convergence was observed between the quantitative and the qualitative results, the operationalization of main concepts and the data reductions will have to be confirmed through subsequent studies. In addition, the sampling method and the criteria used to recruit participants (i.e. European and Canadian Francophones) limit the transferability of the findings to other populations of researchers. Besides DC, it is recognized that social culture, as influenced by language, cultural heritage, political climate, economic conditions, and ethical frames of reference, is a factor influencing the perception of NT applications, regarding the impacts on a set of issues (Gaskell et al. 2005; Kahan et al. 2009; Sechi et al. 2014), including ethical issues (Schummer 2006). Social distinctions have not been addressed here, but these differences regarding social cultures were examined and future works are intended to present these findings.

## Conclusion

Using a mixed-methods design, this study has yielded new empirical data on impact perception, acceptance, and acceptability towards two kinds of drug-delivery nanocarriers in two contexts of use, viewed through the prism of distinctions between sets of disciplinary backgrounds in researchers. It was found that the context of use, the gravity of the disease, and usefulness are important factors that must be taken into account in assessing acceptance and acceptability as regards to medical treatments based on NM. In contrast, the composition of the nanocarrier, though it affects perceived impacts, appears not to influence acceptance or acceptability for a given context. Nor does DC appear to modulate relationships between these variables. During the examination of the acceptability of the carbon nanocarrier to treat seasonal flu, distinct profiles emerged and trends were observed regarding the optimism of NSG researchers and the hesitancies of SSH researchers. The interviews also shed interesting light on the diversity of acceptability judgements in relation to the DC of researchers interviewed about this scenario. Differences relating to fields of expertise and the richness they contribute in establishing a portrait of targeted drug delivery reinforce the view that it is necessary to include perspectives emerging from diverse disciplinary backgrounds. This would encourage the intersection between issues traditionally associated with the NSE on one hand and those associated with the SSH on the other. Finally, since researchers of these sets of disciplinary backgrounds highlight certain potential areas of sensitivity in the development of NTs, based not only on their academic expertise, but also as regard their status of potential user, it appears necessary to continue highlighting and seeking to understand arguments of different kinds, this with a view to pursuing interdisciplinary dialogue on matters of technology development.
